# Extracellular Vesicles: Potential Roles in Regenerative Medicine

**DOI:** 10.3389/fimmu.2014.00608

**Published:** 2014-12-03

**Authors:** Olivier G. De Jong, Bas W. M. Van Balkom, Raymond M. Schiffelers, Carlijn V. C. Bouten, Marianne C. Verhaar

**Affiliations:** ^1^Department of Nephrology and Hypertension, University Medical Center Utrecht, Utrecht, Netherlands; ^2^Department of Biomedical Engineering, Eindhoven University of Technology, Eindhoven, Netherlands; ^3^Department of Clinical Chemistry and Hematology, University Medical Center Utrecht, Utrecht, Netherlands

**Keywords:** regenerative medicine, tissue engineering, extracellular vesicles, exosomes, microvesicles

## Abstract

Extracellular vesicles (EV) consist of exosomes, which are released upon fusion of the multivesicular body with the cell membrane, and microvesicles, which are released directly from the cell membrane. EV can mediate cell–cell communication and are involved in many processes, including immune signaling, angiogenesis, stress response, senescence, proliferation, and cell differentiation. The vast amount of processes that EV are involved in and the versatility of manner in which they can influence the behavior of recipient cells make EV an interesting source for both therapeutic and diagnostic applications. Successes in the fields of tumor biology and immunology sparked the exploration of the potential of EV in the field of regenerative medicine. Indeed, EV are involved in restoring tissue and organ damage, and may partially explain the paracrine effects observed in stem cell-based therapeutic approaches. The function and content of EV may also harbor information that can be used in tissue engineering, in which paracrine signaling is employed to modulate cell recruitment, differentiation, and proliferation. In this review, we discuss the function and role of EV in regenerative medicine and elaborate on potential applications in tissue engineering.

## Introduction

Regenerative medicine aims at the functional restoration of a damaged, malfunctioning, or missing tissue. There are three main approaches in regenerative medicine. The first approach is cell-based therapies, where cells are administered to restore a tissue either directly or through paracrine functions. The second approach is often referred to as classical tissue engineering, and consists of the combined use of cells and a bio-degradable scaffold to form a tissue. Lastly, much progress has been made in material-based approaches, which rely on bio-degradable materials, often functionalized with cellular functions.

The first development in replacing malfunctioning tissues was by transplanting organs, tissues, or cells. Over the course of the last century vast improvements were made in the field of transplantation, starting with bone and cornea transplants at the beginning of the twentieth century, followed by the first kidney transplantation in the 1950s (1–3). As transplantation techniques for other organs developed over the following decades, the limiting factor for these procedures shifted from technical limitations to the supply of suitable organs and tissues. Besides shortage in supply, organ and tissue transplantation have another major drawback: the risk of immune rejection and the required chronic immunosuppression treatment.

In response to these issues, research focused on strategies that allow functional restoration of damaged tissues by cell-free approaches or approaches using autologous cell and tissue sources. Embracing the rapid developments in technology and our understanding of biological processes, the field of regenerative medicine is focusing on a wide array of techniques and approaches to restore tissue function. Suitable approaches depend on the function and environment of the newly generated tissue. For instance, in the replacement of insulin-producing cells in patients with type-1 diabetes, there is little need for load-bearing structures, but rather for structures mimicking the extracellular matrix (ECM) like hydrogels, to retain and stimulate insulin-producing cells (4). Heart valve replacements on the other hand require materials that are able to withstand large forces in addition to high flexibility (5), but due to their direct contact with a patients’ circulation also require the use of materials with high hemocompatibility and low immunogenicity. Utilizing autologous stem-, progenitor-, and tissue-specific cells to restore damaged tissues may bypass the problem of immunogenic responses against these implants. Following recent insights that the structural contribution of stem cells to regenerated tissues is limited, and that rather the stimulation of local healing processes plays an important role (6–9), research has increasingly focused on the paracrine hypothesis, investigating the stimulating factors released by these stem- and progenitor cells, including growth factors, cytokines, and extracellular vesicles (EV). At the same time, major breakthroughs in the field of EV have uncovered roles for EV in many processes including angiogenesis, regulation of immune responses, and ECM remodeling (10–13), which may be of specific interest for tissue engineering. Here, we review the recent developments in regenerative medicine and EV research, and discuss potential therapeutic applications of EV in restoring function in damaged tissues.

## Regenerative Medicine: Cell Therapies

One of the earliest applications of cell therapy was the administration of cells for the reconstitution of blood or bone marrow (14, 15). As a result of developments during the last decades, including improved techniques in both transient and permanent regulation of gene expression, methods of cell isolation and propagation, and improved protocols to regulate differentiation of cells, cell therapies currently play a prominent role in the field of regenerative medicine (16). Cell therapies can directly aid repair by forming new functional tissues, or support tissue repair through paracrine mechanisms, for instance by secreting growth factors, immunomodulatory molecules, and EV. Examples of direct tissue formation by cell therapy are the use of autologous epithelial cells to repair cornea injuries (17), expansion, and transplantation of chondrocytes in cartilage repair (18), or the administration of endothelial colony-forming cells (ECFC) in a murine hind limb ischemia model to increase neovascularization (19). In these studies cell populations were isolated, expanded *ex vivo*, and re-introduced at the site of injury to generate new, functional tissues. The *ex vivo* expansion step allows the use of only limited amounts of tissue and the proper characterization of isolated cells. Adverse effects as dedifferentiation and induction of senescence are great challenges adhered to this approach (20). For instance, *in vitro* passaging of mesenchymal stem cells (MSC) results in cell enlargement, differentiation, and decrease in proliferation within 10 passages (21), and causes a strong response to micro-environment stiffness, affecting cell morphology, and function (22). Progenitor cells from aged or diseased donors show decreased proliferation, prevalence, as well as functionality (23–25). Despite these challenges, promising results have been achieved, for instance in treatment of patients with severe autoimmune diseases with hematopoetic stem cell transplantation (26).

It has become increasingly apparent that a more supporting role, employed by secretion products of stem and progenitor cells is responsible for many of the observed effects of stem cell therapies (6–9). These paracrine factors secreted by stem- and progenitor cells, like growth factors and cytokines, are of major interest to discover new therapeutics that stimulate local tissue regeneration for the use in tissue engineering as well [reviewed in Ref. (27, 28)].

## Tissue-Engineering: (Bio-)Engineered Support

Repair of damaged tissue requires not only the presence of cells capable of restoring the damaged structure, but requires a microenvironment that promotes appropriate tissue regeneration as well. In addition, cells need to be guided to form a structure of the appropriate size and shape, and in many cases (for instance in bone or cartilage repair, as well as in cardiovascular substitutes), require structural support. In a healthy tissue, the ECM plays a key role in guiding and regulating these processes, whereas in damaged tissue, the ECM is often absent, damaged, or functionally impaired. To address this problem and allow *in situ* regeneration, structures that (temporarily) provide the requirements for cell retention and tissue regeneration are employed and are referred to as scaffolds.

Scaffolds can either be of natural origin, such as decellularized ECM or modified elastin- or collagen gels, or of synthetic origin, such as synthetic hydrogels or porous polymer scaffolds. Using decellularized ECM from xenogenic or allogenic donors provides scaffolds that are most similar to the natural extracellular environment. Use of decellularized matrices is a promising technique, which yields biocompatible scaffolds with appropriate physical and biological properties. Many ECM components, as well as growth factors, are often conserved and can aid in proper regeneration of functional tissues (29). To decrease the risk of immune responses against antigens in these scaffolds, as well as the potential transfer of pathogens, a combination of enzymatic, physical, and chemical treatments is used to remove cellular components from the tissue (29). Decellularized matrices have been used for tissue engineering of several tissues, including heart valves (30), vascular grafts (31), and trachea (32). However, use of decellularized matrices has several disadvantages. Acquiring and isolating of appropriate tissues, followed by decellularization protocols, can be a relatively time-consuming and expensive procedure, and incomplete decellularization or antigen removal can result in immune reactions against grafts (33). Cell seeding of decellularized matrices can be technically challenging due to structural dimensions and porosity. Furthermore, control over the exact content of the matrices is limited due to donor variation, and despite pretreatment still there exists the risk of transfer of pathogens. In order to create scaffolds in a safe, reproducible, affordable, and controlled manner, extensive research is ongoing on the production of artificial porous scaffolds, exploring various production techniques and materials (34).

Artificial porous scaffolds should meet specific requirements to allow homing of appropriate cell populations. Ideally, a synthetic scaffold temporarily provides the required support and micro-environment, is bio-degradable and eventually replaced by autologous ECM. For cells to be able to migrate or be seeded in the scaffold and allow an environment with proper supply of nutrients, a porous structure is required (35). There are several techniques to generate porous scaffolds, including solvent casting, forming emulsions before polymerization, gas foaming, as well as binding of polymeric fibers by chemical treatment or heating (36–39). Using these techniques in generating scaffolds with consistent porosity in complex shapes, containing areas of varying thickness and materials, is technically challenging. Currently, the most commonly used technique in generating porous synthetic scaffold is electrospinning, which allows the generation of constructs with complex geometry, consisting of combinations of fiber types in both mixed and layered patterns (40). Bio-degradable polymers used in electrospinning include poly(ε-caprolactone) (PCL), poly(glycolic acid) (PGA), poly(hydroxy alkanoate) (PHA), and poly(lactic acid) (PLA). Mixing fiber types in specific patterns allows modulation of degradability, strength, and biological activity of scaffolds (41).

**Figure 1:**
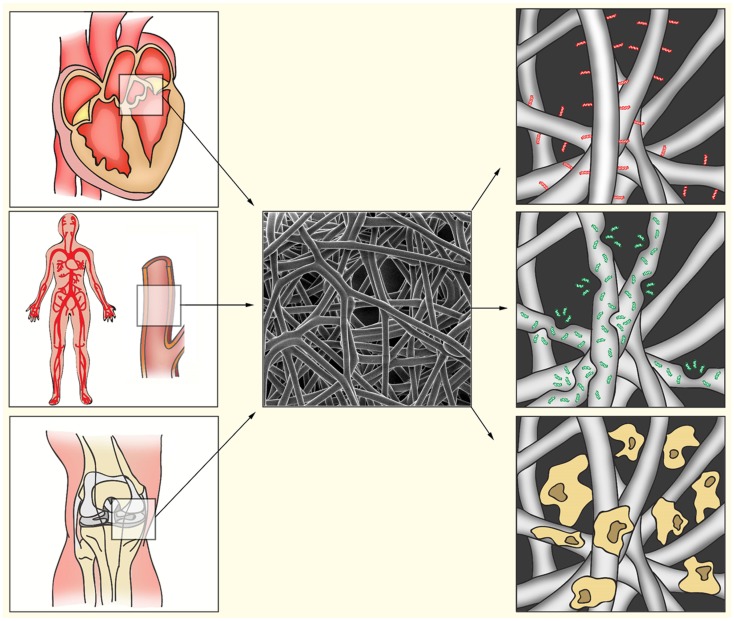
**Bio-activated artificial scaffolds**. Electrospinning allows formation of constructs with a variety in shapes, sizes, and tissue strength. This allows the production of constructs for a variety of tissues (left). Electrospun fibers (middle) can be bio-activated by coating of the fibers with proteins or peptides (right, red). Incorporation of bio-active components into the fibers will result in gradual release during fiber degradation (right, green). After electrospinning, fibers can also be pre-seeded with appropriate cell populations to induce ECM production, angiogenesis, or immunomodulation (right, yellow).

Electrospun scaffolds can be pre-seeded with autologous cells, which may be re-programmed, differentiated, and expanded *in vitro*, and can then be directly implanted or incubated in a bioreactor until the electrospun meshwork is fully degraded and replaced with ECM (5). Alternatively, scaffolds can be implanted without pre-seeding, allowing *in situ* recruitment of autologous cells and circumventing the expensive, time-consuming, and challenging process of cell isolation and expansion *in vitro*. Incorporation of bio-active molecules into the scaffold may be used to recruit proper cell populations, modulate the immune response, or guide cells to differentiate (Figure 1). For instance, ECM-derived peptides like the integrin recognition site peptide Arg-Gly-Asp (RGD) enhance cell adhesion and cell viability in scaffolds (42, 43), whereas coating with type I collagen-mimetic peptide enhances the migration, proliferation, and osteogenic differentiation of MSC (44). Scaffolds can also be designed to release peptides, proteins, or cytokines during degradation, or by coating fibers with a mixture of these bio-active molecules in a bio-degradable substance like fibrin or gelatin. For example, gradual release of vascular endothelial growth factor (VEGF) – a hypoxia-regulated growth factor that plays a key role in angiogenesis – and platelet-derived growth factor (PDGF) promoted endothelialization and smooth muscle cell ingrowth in electrospun scaffolds (45). Release of stromal cell-derived factor (SDF)-1α – a chemokine that is up-regulated in tissue damage and hypoxia, attracts hematopoietic stem cells, and induces endothelial progenitor cell (EPC) recruitment – by electrospun poly(lactic-co-glycolic acid) (PLGA) scaffolds reduced mast cell degranulation, and increased angiogenesis and decreased fibrosis (46). Coating of interposition grafts with SDF-1α combined with the ECM component fibronectin (47), or treatment with VEGF (48) has been reported to enhance graft endothelialization.

Many of the bio-active compounds used in these approaches act as paracrine factors in natural healing processes, or on the secretome of stem- or progenitor cell populations that induce local tissue regeneration *in vivo* (27). EV constitute a part of the secretome that also play an important role in local induction of tissue regeneration. For example, cardioprotective effects of conditioned medium from MSC in ischemia/reperfusion injury were shown to be mainly mediated by EV (49). Given the previous successes of paracrine factors in tissue engineering, these mediators of intercellular communication could also be of interest in the field of regenerative medicine.

## Extracellular Vesicle Characteristics

**Figure 2:**
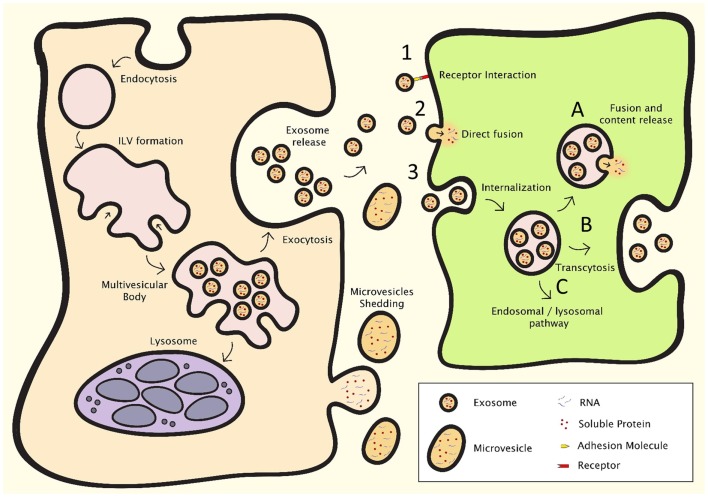
**EV formation (left) and intercellular communication (right)**. After endocytosis, intraluminal vesicle formation occurs in the late endosome, resulting in the formation of the multivesicular body (MVB). The MVB can either fuse with the lysosome, resulting in breakdown and recycling of its contents, or fuse with the plasma membrane, resulting in the release of the intraluminal vesicles, which are then deemed exosomes. Microvesicles shed directly from the plasma membrane. Intercellular communication can occur through three major processes: (1) direct interaction of ligands expressed on the surface of EV with receptors on the cell membrane, (2) direct fusion of the EV with the cell membrane, resulting in the release of the content of the EV, or (3) internalization through the endocytotic pathway, which can result in (A) fusion of the EV with membrane of the endosome, resulting in content release, (B) transcytosis, or (C) degradation through the lysosomal pathway.

Extracellular vesicles are lipid membrane vesicles, containing a variety of RNA species (including mRNAs, miRNAs), soluble (cytosolic) proteins, and transmembrane proteins presented in the appropriate, and functional orientation (50–52). EV play a role in many processes, including intercellular communication, recycling of membrane proteins and lipids, immune modulation, senescence, angiogenesis, and cellular proliferation and differentiation (10, 13, 52–56). Cells release several types of vesicles with different physiological properties, content, and function, as a result of their different mechanisms of generation, and include exosomes, microvesicles, and apoptotic bodies (57). In the EV research community, a full consensus in terminology and classification of vesicles is yet to be achieved (58). In the past, vesicle nomenclature was mainly based on the tissue of their origin. More recently, the field has started to shift toward a terminology that focuses rather on the mechanisms of generation of these vesicles. Vesicles in the first category, exosomes, originate in multivesicular bodies (MVB) (Figure 2, left). When MVB fuse with the plasma membrane, the intraluminal vesicles are released from the cell and are from thereon referred to as exosomes. Exosomes are reported to be between 40 and 150 nm in size, with a density ranging from 1.09 to 1.18 g/ml. The most common markers used are tetraspanins such as CD9, CD63, CD81, and CD82, lipid raft markers Flotillin-1 and -2, as well as Alix and Tsg101. Other markers that are used are heat shock proteins, MHC molecules, various components of the ESCRT complex and proteins of the Rab protein family (50, 51, 59–61). Microvesicles are shed directly from the plasma membrane and can be a lot larger than exosomes (50–1000 nm) (62). There is, however, an overlap in size between these two populations. Microvesicles also contain mRNAs and miRNAs, as well as soluble and transmembrane proteins. Like exosomes, microvesicles are able to transfer functional genomic and proteomic content to target cells (63, 64). Apoptotic bodies originate at the cell membrane as cells undergo apoptosis. Even though these vesicles are of interest in biomarker research, and have been shown to have effects on other cells, research on these vesicles in intercellular communication is limited (65–67). Furthermore, vesicular cell-derived microparticles with biological functions have been described (68–70). However, most descriptions of microparticles are heterogeneous with regard to the isolated biomaterials or refer to characteristics of non-cell-derived compounds, and depending on the protocols used these microparticles may contain exosomes, microvesicles, apoptotic bodies, or varying combinations of these vesicle populations. Generally, the term EV is used when discussing exosomes or microvesicles, or a combination of these vesicle populations, depending on isolation techniques. However, due to the technical limitations of current isolation techniques, samples may occasionally also contain apoptotic bodies and protein aggregates.

The first report of a cellular function of exosomes was the shedding of the transferrin receptor by maturing reticulocytes (55, 71). Pan and Johnstone showed that removal of this receptor from the cell membrane occurred through endocytosis, followed by formation of intraluminal vesicles (forming the MVB), which were released when the MVB fused with the cell membrane. After this discovery, it was believed that the exosome pathway was mainly involved in cell homeostasis, by secreting cellular waste (72). Not until a study of Raposo et al. for the first time showed an immunological role for exosomes, the stimulation of CD4^+^ T-cells by EBV-transformed B-cells in an antigen specific manner, did researchers begin to explore additional functions (12). Primarily being studied in the context of immunology, exosomes were increasingly considered potential mediators for intercellular communication. However, it was only after the discovery that exosomes are able to transfer functional mRNAs and miRNAs from one cell to another, that the field gained its full momentum (52, 73). Microvesicles have also been reported to transfer functional mRNAs and miRNAs to cells (66, 67).

Extracellular vesicles can communicate with target cells through several mechanisms (Figure 2, right). Firstly, transmembrane proteins on the EV membrane can interact with receptors on the cell membrane. These receptor–ligand interactions can then activate signaling cascades to affect target cells. EV can also fuse with their target cells to release their cargo, either by direct fusion with the cell membrane or by endocytosis, after which mRNAs, miRNAs, and proteins are released into the cytosol. Fusion of EV with target cells can either occur directly at cell membrane, or after endocytosis. After fusion, mRNAs transferred by EVs can be translated in to protein, and delivered miRNAs inhibit mRNA translation and affect cellular processes. The cargo and function of EV depends on their producing cells, and it has been shown that also cellular stress affects EV content, suggesting that intercellular communication through EV is a dynamic system, adapting its “message” depending on the condition of the producing cells (50–52, 74).

## Extracellular Vesicles in Regenerative Medicine

**Figure 3:**
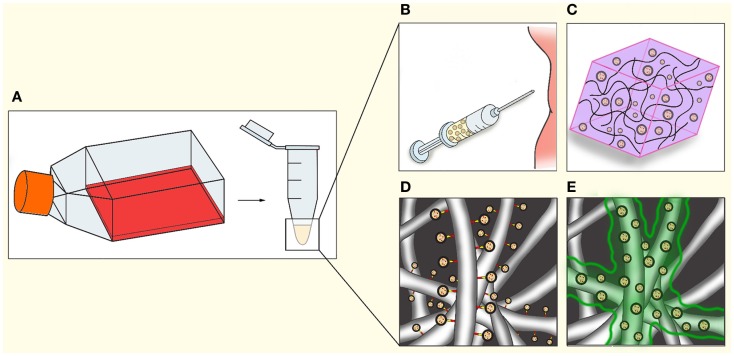
**Applications of EV in regenerative medicine**. After isolation **(A)**, EV could be utilized in regenerative medicine through a number of methods, either separately or in combination with cells or other therapeutics. **(B)** Direct injection into tissue or circulation. **(C)** Mixing of EV in hydrogels. **(D)** Coating electrospun fibers indirectly via chemical linkers, antibodies, or specific tags engineered on to the EV. **(E)** Coating of electrospun fibers with bio-degradable gels such as fibrin, resulting in gradual release during gel degradation.

Extracellular vesicles are able to affect cell phenotype, recruitment, proliferation, and differentiation in a paracrine manner. These paracrine effects of EV have a potential benefit in regenerative medicine. EV can be incorporated in regenerative therapies, for example by (co-)injection, mixing with hydrogels, or coating scaffolds with EV using fibrin gels or specific linkers (Figure 3). Here, we will discuss the role of EV in essential processes in regenerative medicine: cell viability, immune responses, ECM interaction, and angiogenesis.

### Cell senescence, viability, and proliferation

Prevention of cell death and cell senescence is vital in optimizing efficiency of regenerative medicine, both in cell therapies as well as in tissue engineering (75). Cell senescence depends on both the cell source and the environment to which cells will be introduced. Bone marrow-derived MSC from aged donors show increased senescence, and decreased proliferative potential (76, 77), and uremic toxins promote cell senescence (78). Pretreatment of progenitor cells such as MSC affects cell senescence as well. For example, long-term *in vitro* expansion of MSC induces senescence, and reduces differentiation potential (79, 80).

Extracellular vesicles may affect cell senescence, proliferation, and cell survival. We recently demonstrated that endothelial cell-derived exosomes induced angiogenesis by inhibition of cellular senescence, and that transfer of miR-214 downregulated ataxia telangiectasia mutated (ATM) expression in recipient cells, resulting in decreased cellular senescence (13). Human umbilical cord MSC-derived microvesicle treatment suppressed cisplatin-induced apoptosis, and resulted in increased cell proliferation through regulation of the ERK 1/2 and MAPK pathways, both *in vitro* and *in vivo* (81). EV derived from human cardiac progenitor cells contain anti-apoptotic miRNAs, miR-210 and miR-132, and treatment with these EV in a myocardial infarction resulted in decreased cardiomyocyte apoptosis (82). Similarly, bone marrow MSC-derived exosomes were able to decrease apoptosis and increase cell proliferation in an acute kidney injury model, and the authors hypothesized that this was the result of exosome-mediated RNA transfer (83). Similar results were obtained by Bruno et al., who showed that administration of MSC-derived microvesicles decreased apoptosis in an acute kidney injury model and *in vitro* in cisplatin treated human epithelial cells, through up-regulation of anti-apoptotic genes and down-regulation of several apoptotic genes (84). Further *in vitro* studies showed that cardiomyocyte protection by MSC is partially mediated by transfer of miR-221 in microvesicles, resulting in reduced caspase activity after ischemic injury (85). Certain EV have also been shown to increase cell proliferation. Tumor-derived EV were reported to induce proliferation in a variety of tissues (86–88). MSC-derived EV have also been found to increase proliferation: bone marrow MSC-derived exosomes induced proximal tubular epithelial cell proliferation in an acute kidney injury model (89), and umbilical cord MSC-derived exosomes increased *in vitro* skin cell proliferation as well as migration after heat-stress, through Wnt signaling by trafficking of Wnt4 (90). Interestingly, Zhang et al. also observed that treatment with these vesicles in a rat skin burn model resulted in accelerated epithelialization (90). Exosomes derived from tubular epithelial cells stimulated with hypoxia activated fibroblasts through TGF-β1 signaling, resulting in increased fibroblast proliferation, which could aid in acceleration of tissue repair (91). These studies indicate that EV play a role in local tissue repair through regulation of cell proliferation.

The capacity of EV to regulate cell senescence, apoptosis, and proliferation, parameters that greatly affect tissue engineering and cell therapy outcome, suggest therapeutic potential in regenerative medicine. Indeed, MSC-derived vesicles show positive effects on tissue repair through various pathways, even reducing apoptosis as a result of ischemic injury (92). This is of interest, since ischemia in larger tissue-engineered constructs is a substantial issue (93).

### Angiogenesis

Tissue engineering of large tissues requires proper vascularization for sufficient supply of nutrients and oxygen, and draining of cellular waste. Since tissue-engineered constructs thicker than 100–200 μm already run in to problems in respect to oxygenation, nutrient supply, and removal of waste products, controlled vascularization of neo-tissue is vital (93). Strategies to induce vascularization include addition of endothelial (progenitor) cells, engineering vasculature, as well as the use of paracrine factors (93–95). Several studies on cancer-derived EV demonstrated their role in tumor angiogenesis through a variety of pathways, including cell cycle-related mRNAs, several major intracellular kinase pathways, transfer of miRNAs, and by carrying pro-angiogenic cytokines (96–100). EV from endothelial cells have also been demonstrated to induce an angiogenic program in target endothelial cells *in vitro* and *in vivo* both through Notch-dependent tip-cell formation and induction of a pro-angiogenic program in parallel to miR-214-dependent repression of senescence (13, 101). EV from other cell types have been demonstrated to stimulate *in vitro* and *in vivo* vessel formation by endothelial cells as well. For example, adipose MSC-derived EV, which could be increased in function and number by PDGF stimulation (102), as well as bone marrow MSC-derived EV, promoted angiogenesis in a rat myocardial infarction model (103). In the latter model, hypoxic stimulation of the EV-producing cells was required to obtain functional EV. Similar effects of hypoxia were observed in microvesicles from human umbilical cord MSC, which promote angiogenesis *in vitro* as well as *in vivo* in a rat hindlimb ischemia model (103, 104). These findings underline the importance of culturing conditions of their producing cells on EV content (74). Cantaluppi et al. showed that EPC-derived microvesicles increase endothelial cell proliferation, migration, and vessel formation *in vitro* by transfer of pro-angiogenic miRNAs, miR-126 and miR-296. These EPC microvesicles also increased vascularization of islet endothelium and β-cells transplanted in SCID mice (105) and, in a SCID mouse hind limb ischemia model increased capillary density, enhanced limb perfusion, and reduced injury after 7 days (106). A study by Sahoo et al. in 2011 showed that exosomes isolated from CD34^+^ mononuclear cells increased endothelial cell viability, proliferation and tube formation *in vitro*, and stimulated angiogenesis *in vivo* in both matrigel plug- and corneal assays, and that the pro-angiogenic effect of these cells was mainly through these EV (107).

Overall, different types of EV appear to be able to induce angiogenesis through a variety of pathways, and through transfer of mRNA, miRNAs, and proteins, underlining their potential in tissue engineering.

### Extracellular matrix interactions

The ECM plays a major role in tissue engineering, providing shape and strength to the newly formed tissue as well as a site for interactions with and guidance of cells. Both ECM architecture and molecular composition are determinants for cell recruitment, retention, and differentiation, and thus the final local cell phenotype. In tissue engineering strategies using bio-degradable scaffolds, the load-bearing and cell retaining function of the scaffold will have to be fulfilled by the locally produced ECM after the scaffold is degraded. EV are able to influence ECM composition through direct ECM interactions, or by interacting with ECM-producing cells.

Extracellular vesicles express adhesion molecules, including members of the immunoglobin superfamily and integrins. Exosomes derived from B-cells, endothelial cells, and dendritic cells, express ICAM-1 (74, 108, 109), and endothelial cell-derived exosomes express CD44, CD166, PECAM, and B-CAM (74). Reticulocyte-derived exosomes have been shown to bind to fibronectin via integrin α4β1 (110). B-cell-derived exosomes contain β1 and β2 integrins, which were able to bind to collagen-1, fibronectin, and TNF-α activated fibroblasts (108). Exosomes derived from dendritic cells have also been reported to contain integrins (111). These studies show that EV may not only bind to and interact with cells, but also bind to various ECM components. It has been suggested that EV could adhere to the ECM to form a gradient or potential reservoir that could be released in case of inflammation or ECM degradation (108).

Besides molecules responsible for ECM interaction, EV have also been shown to express ECM-remodeling proteins, like matrix metalloproteinases (MMPs), which can degrade collagens, elastin, fibronectin, and laminin. These processes are important in ECM re-structuring, as well as cytokine release, angiogenesis, and cell migration (112, 113). For example, human fibrosarcoma and melanoma cell-derived exosomes contain both full length and proteolytically processed MMP14, shown to be enzymatically active since these exosomes activated pro-MMP2 resulting in the degradation of both collagen-1 and gelatin (114). Cardiomyocyte progenitor-derived exosomes expressed enzymatically active MMP2, as well as MMP-activator EMMPRIN (115). EMMPRIN has also been found on CD8^+^ T-cell microparticles, which have been shown to induce fibrolytic activation in hepatic stellate cells (70). Madin-Darby canine kidney cells (MDCK) that have undergone epithelial to mesenchymal transition (EMT) showed an increase in MMP1, -14, and -19 expression in their exosomes, as well as several integrins (116). Additionally, EV can also stimulate MMP production in target cells. Keratinocyte-like cells are able to stimulate MMP1 expression in dermal fibroblasts through transfer of several 14-3-3 isoforms by EV (117). Furthermore, monocyte and T-cell-derived microparticles are able to induce production of MMP-1, MMP-3, MMP-9, and MMP-13 in fibroblasts (68). Thus, EV can influence MMP abundance and activity on several levels.

Extracellular vesicles also have the ability to contribute to ECM strength. Members of the lysyl oxidase family crosslink collagens and elastin, increasing ECM load-bearing properties. Lysyl oxidase treatment of tissue-engineered cartilage constructs results in increased stiffness and enhanced cartilage integration, and lysyl oxidase-like 2 induces angiogenic sprouting through interacting with collagen-4 in the basal membrane (118, 119). Lysyl oxidase was shown to be enriched in exosomes derived from hypoxic glyoma cells (98) and lysyl oxidase-like 2 in endothelial cells (74). Interestingly, exosomes from hypoxic endothelial cells also showed increased abundances of the ECM components fibronectin, collagen-4 and -12 subunits, and perlecan, suggesting a hypoxia-mediated role in focal ECM modification by exosomes (74). EV are also able to affect local ECM production. Borges et al. found that upon hypoxic stimulation, epithelial cells stimulate fibroblasts through exosome-mediated TGF-β1 signaling, resulting in increased collagen-1 production (91) and suggesting an exosome-mediated response resulting in local tissue repair. The effects of EV on both ECM production and remodeling could be of use in the steering of *in situ* ECM formation.

### Immunomodulation

Modulating immune responses is vital in tissue engineering. The type and severity of the immune response against an implant depends on several factors including injury from surgery, the (bio)materials used, location of the graft, and the condition of the patient (120). An excessive or inappropriate immune response could result in damage, encapsulation or rejection of a tissue-engineered construct. On the other hand, immune responses are potent triggers for regenerative processes, including cell recruitment, proliferation, and angiogenesis, which are key to the success of *in situ* tissue engineering (121).

When transplanting a tissue-engineered construct, the innate immune response consists of the acute and the chronic phase. The acute immune response is an immediate reaction against foreign structures, such as certain (bio)materials. An influx of neutrophils and macrophages induces the release of inflammatory cytokines, which results in local inflammation and the recruitment of additional immune cells. Cross-talk between macrophages and T-cells, as well as environmental cues, regulate a shift in macrophage sub-types in to either M1 (inflammatory), or the M2 (anti-inflammatory, regenerative) subtype (122). M1 macrophages promote recruitment of inflammatory immune cells, and release ECM-degrading proteins to allow quick migration through inflamed tissues. As the subtype of macrophages shifts to M2, pro-inflammatory cytokine release is inhibited, angiogenic stimulation is increased, and local fibroblasts are activated in order to produce and restore the ECM. Long-term inflammation results in a foreign body response (FBR) in which case a foreign tissue is encapsulated by a fibrous, barely vascularized connective scar-like tissue (123). An antibody-mediated immune response against allografts or tissues seeded with non-autologous cells could result in rejection of a graft. These findings underline the importance of tuning the immune response in tissue engineering: sufficient to induce vascularization, cell recruitment, and ECM production, while preventing fibrosis, tissue damage, and FBR.

The modulatory role of EV in innate immune responses could prove beneficial in tissue engineering. MSC-derived exosomes induced an M2-like phenotype in monocytes *in vitro*, resulting in polarization of activated CD4 T-cells to regulatory T-cells (124). Additionally, tumor-derived exosomes have been shown to induce a shift toward an activated M2 phenotype (125), as well as an M1 phenotype (126). Furthermore, EV can play a role in the suppression of allograft rejection. Autologous regulatory T-cell-derived exosomes postponed allograft rejection in a rat kidney transplantation model (92). Immature dendritic cell-derived exosomes induced allograft tolerance in a cardiac allograft mouse model (127), as well as in a rat intestinal transplantation model (128) by increasing regulatory T-cell populations.

Mesenchymal stem cells themselves have been a tool of interest for their immunosuppressive capacities, inhibiting B- and T-cells, natural killer cells, macrophages, and dendritic cells (129–131). Accordingly, MSC-derived exosomes promote secretion of anti-inflammatory cytokines, and contain an array of tolerogenic molecules (132), and administration of MSC-derived exosomes in a myocardial ischemia/reperfusion injury model showed a significant reduction of local and systemic inflammation after 24 h (133). In a renal ischemia-reperfusion model in rats, MSC-derived microvesicles administered to the caudal vein inhibited inflammation as well as renal fibrosis (134). Indeed, a systematic literature study of MSC-derived EV revealed that modulation of EV responses, as well as repair of organ injury and suppression of tumor growth in preclinical studies, shows therapeutic potential (135).

The potential immunomodulatory role of EV may be relevant for regenerative medicine by steering vascularization, cell recruitment, and ECM formation, as well as the prevention of tissue damage, and FBR.

### Extracellular Vesicles potential

All in all, EV show great potential for a role in regenerative medicine because of their role in cell recruitment, differentiation, and immunomodulation (Table 1). Many of these functions of EV may also be combined with other regenerative strategies as their effects on nutrient and oxygen supply, immune responses, and cell viability and senescence may benefit efficacy of approaches in regenerative medicine, such as cell therapies or *in situ* tissue engineering (27, 75, 93). Given the role of EV in processes that greatly affect tissue regeneration, further studies in EV-mediated paracrine signaling and exploration of new methods to utilize EV or components thereof is warranted and may lead to the discovery of novel regenerative therapeutics, as well as methods to improve current techniques.

**Table 1:** **Functional relevance of EV in regenerative processes**.

Process	Contribution	Reference
Cell senescence, viability and proliferation	Inhibition of cellular senescence	13
	Inhibition of apoptosis	81–85
	Increased cell proliferation	81, 83, 86, 89–91
Angiogenesis	Transfer of pro-angiogenic proteins	96
	Transfer of pro-angiogenic miRNAs	105
	Notch signaling	101
	Inhibition of endothelial senescence	13
ECM interactions	Formation of signaling reservoir in ECM	108
	ECM remodeling through MMPs	68, 70, 115–117
	ECM crosslinking by lysyl oxidases	74, 98
	Inducing ECM production	91
Immunomodulation	Steering M1-M2 macrophage phenotype	124–126
	Increasing regulatory T-cell population	128
	Decreasing graft rejection	85, 127, 128
	Promoting anti-inflammatory cytokine secretion	132

*An overview of potential roles of EV in regenerative medicine*.

## Applications of Extracellular Vesicles in Regenerative Medicine

Even though, the existence of EV was discovered decades ago, interest in their role as paracrine factor was only relatively recently sparked. Much remains unknown about the pathways that determine the content of EV, and many tissue-specific functions of EV remain to be uncovered. Future studies will provide new insights in EV function and biogenesis, and reveal the roles of proteins and miRNAs in EV function. EV are important components of the secretome involved in intercellular communication, of which content and function can change depending on the conditions of the vesicle producing cells (74, 91, 102–104). Therefore, changes in EV content upon stimulation of producing cells with conditions relevant in development, tissue regeneration, and wound repair may reveal new pathways and insights in intercellular signaling that play key roles in these conditions. Altogether, these qualities make EV an interesting target for the potential discovery of new therapeutics in regenerative medicine.

### Extracellular Vesicles as Therapeutics

Extracellular vesicles from specific cell types and conditions have positive effects on regeneration in many tissues (136). It has also been observed that certain EV display multiple functions. For instance, MSC-derived EV are able to steer cell viability, proliferation, angiogenesis, and immune responses (81–83, 103, 104, 124, 132). Harnessing the paracrine effects of stem- and progenitor cells without having to administer living, replicating, potentially pluripotent cell populations is an advantage in regard to safety, regulation, and complexity.

However, there are challenges to overcome. The current golden standard in isolation of functional EV remains ultracentrifugation (58), which is a time-consuming and costly procedure that requires a large amount of cells. Although faster commercial reagents are available, which isolate higher yields of EV, these products still require optimization in specificity as they have been reported to also precipitate non-EV contaminants such as lipoproteins (137). Despite decades of research, EV cargo trafficking pathways have not completely been elucidated, and therefore control over the content of EV, and unspecific additional effects, is limited. Research in both biogenesis of EV, as well as techniques for engineering for artificial alternatives for EV is therefore warranted.

### Extracellular Vesicles modification

The concept of developing synthetic alternatives for EV is motivated by the challenges that have been described above: the ability to form synthetic EVs would allow control over these elements, which would facilitate clinical translation. The approach could vary from modulation of biological EV synthesis to a purely synthetic production method. In the first approach, the EVs are still harvested from cells, but the producing cells have been engineered to enrich EVs with tags or therapeutic molecules. Incorporated tags could be used to assist in EV purification, or for targeting toward specific tissues, cells, or synthetic scaffolds. Also, the therapeutic payload can be enriched by overexpression of specific RNAs or proteins (138, 139).

More control over EV content can be achieved by a semi-synthetic approach, which is based on techniques used in the therapeutic enveloped virus-field. Here, the viral envelope is solubilized in a high critical micelle concentration detergent. As a result the proteins and lipids that are part of the envelope are present in micelles that can be separated from the viral capsid. By removing the detergent, the envelope is reconstituted, and “virosomes” are formed (140). Translating this approach to EVs may improve the control over the composition of the bilayer, which additionally can be enriched with desired molecules, as well as during the reconstitution step, offering full control over the encapsulated (therapeutic) compounds in the aqueous core. At the same time, the naturally encapsulated molecules are removed.

### Synthetic Extracellular Vesicles

The semi-synthetic approach still relies on the biological production of vesicles. The power of synthetic strategies lies in the scalability of the process. The minimal EV mimic is already on the market and is known as liposomes (141). Liposomes consist of a phospholipid bilayer around an aqueous core, and have been investigated as therapeutic delivery systems over the last 40 years. Therapeutic liposomes tend to be around 100 nm in size and have a lipid composition that allows them to circulate for prolonged periods in the blood stream. Generally, therapeutic liposomes are prepared in batches that vary between liters to hundreds of liters in size, with a colloidal stability of several years, even in solution. Still the translation of liposome technology to mimic EVs has some obstacles to overcome. For instance, the lipid and protein composition of EV, which may be important for their cellular interactions, is often complex, and the current production process of liposomes involves simple synthetic lipid mixtures without other components within the bilayer. However, liposomes have been successfully equipped with targeting ligands (such as antibodies) and a variety of therapeutic payloads including biologicals (142). These characteristics are several orders of magnitude away from the current state of the art in the EV field, but do illustrate the potential value of synthetic EV.

## Conclusion

Over the past decades, it has been shown that EV play a regulatory role, and have modulatory potential, in many biological processes. EV show great potential for therapeutics, biomarker research, and even alternatives to stem-cell-based therapies which rely on paracrine effects. These new approaches have great potential for the support of endogenous repair, including enhancements of existing regenerative medicine approaches. This potential merits further research in the potential of EV, as well the study of new techniques to produce and utilize engineered EV.

## Conflict of Interest Statement

Raymond M. Schiffelers is the CSO of Excytex, a company developing tools for extracellular vesicle research. The other co-authors declare that the research was conducted in the absence of any commercial or financial relationships that could be construed as a potential conflict of interest.
